# Supplementary material for: Optoelectronic and Electrothermal Properties of Transparent Conductive Silver Nanowires Films
Source: Nanomaterials (Basel). 2019 Jun 21;9(6):904. doi: 10.3390/nano9060904 (PMC6631837; doi:10.3390/nano9060904)
Supplement: Supplementary file 1 [file nanomaterials-09-00904-s001.pdf]

Supporting Information

Optoelectronic and Electrothermal Properties of Transparent Conductive Silver Nanowires Films

Yuehui Wang [1,*], Dexi Du [1,2], Xing Yang [1,2], Xianfeng Zhang [1,*] and Yuzhen Zhao [3]

1 Zhongshan Institute, University of Electronic Science and Technology of China, Zhongshan 528402, China; dudexi_work@foxmail.com (D.D.); shirleywyh@126.com (X.Y.)

2 School of Materials and Energy, University of Electronic Science and Technology of China, Chengdu 610054, China

3 Department of Materials Science and Engineering, Tsinghua University, Beijing 100084, China; zhaoyz@mail.tsinghua.edu.cn

* Correspondence: wangzsedu@126.com (Y.W.); zhangxf07@gmail.com (X.Z.); Tel.: +86-760-8832-5402 (Y.W. & X.Z.)

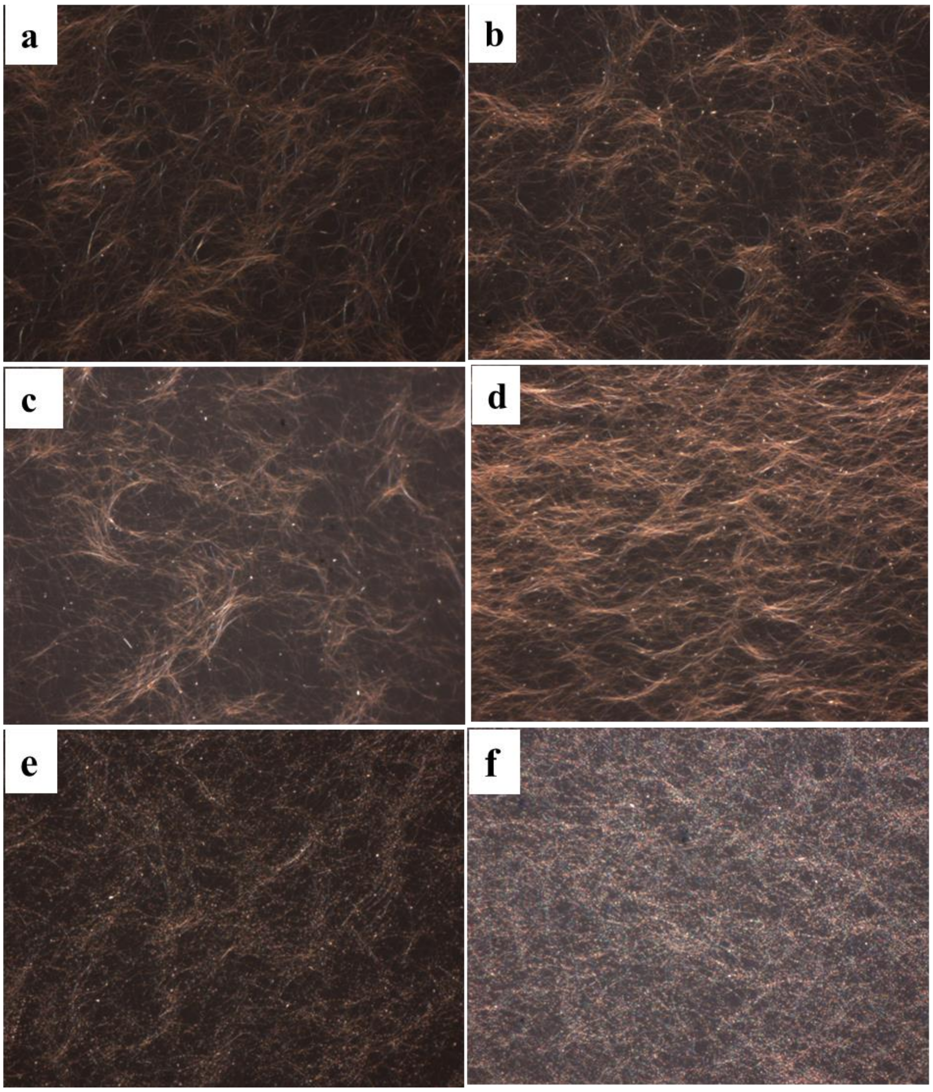

Figure S1. Optical microscope photos of AgNW films treated at (**a**) 25, (**b**) 100, (**c**) 150, (**d**) 170, (**e**) 200, and (**f**) 250 °C for 20 min. (1000× magnification).

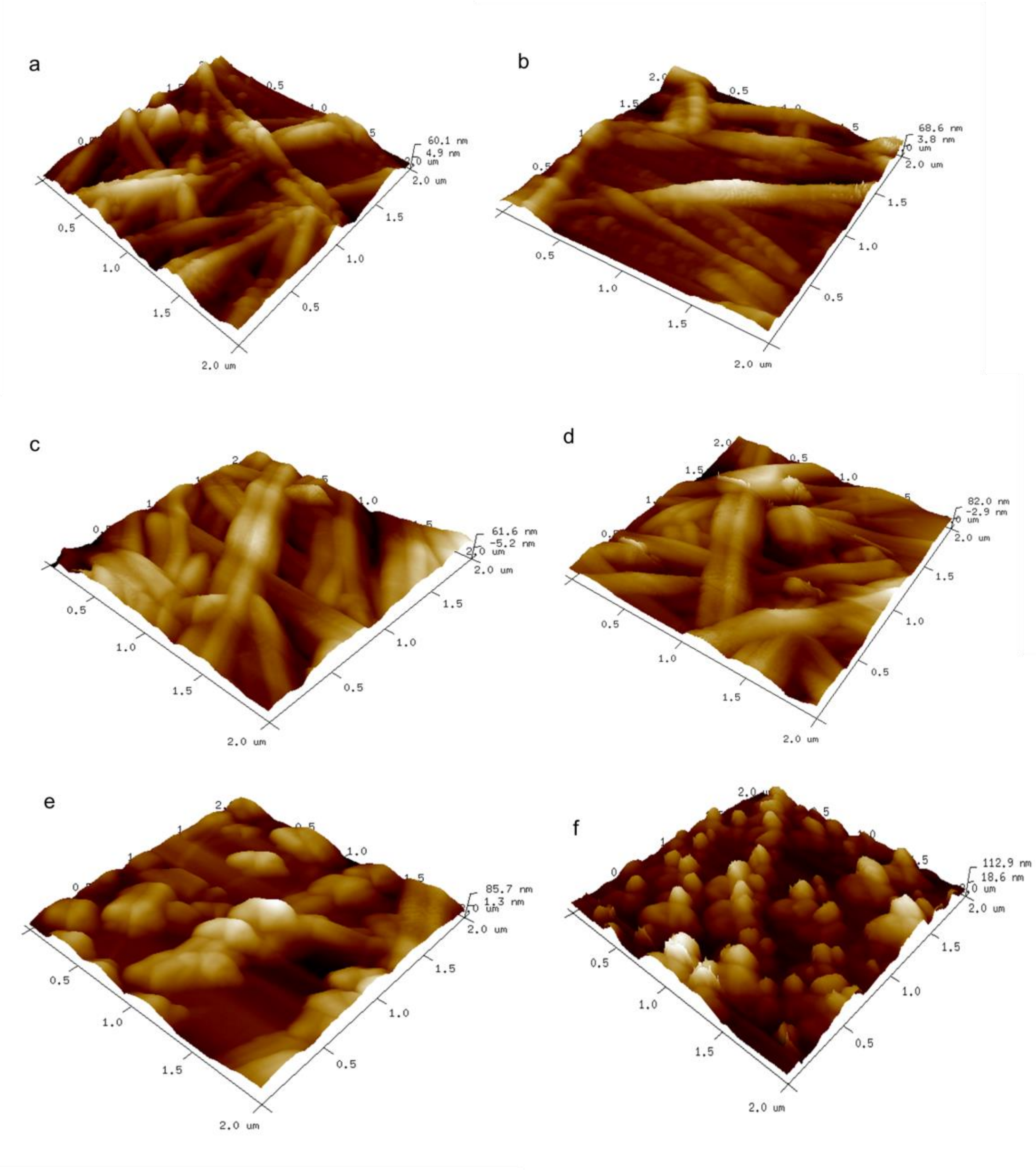

Figure S2. 3D AFM images of the AgNWs films treated at (**a**) 25, (**b**) 100, (**c**) 150, (**d**) 170, (**e**) 200, and (**f**) 250 °C for 20 min.
